# Effect of titanium dioxide nanotubes incorporated into conventional glass ionomer cement on *L. acidophilus*

**DOI:** 10.1590/1807-3107bor-2025.vol39.059

**Published:** 2025-06-02

**Authors:** Layse de Góis SENA, Maria Davoli MEYER, Mariana Gallante RICARDO, Isaac Jordão de Souza ARAÚJO, Julia Puppin RONTANI, Vanessa Arias PECORARI, Elizabeth Ferreira MARTINEZ, Lucas Novaes TEIXEIRA, Francisco Humberto NOCITI-JUNIOR, Paulo Noronha LISBOA-FILHO, Kamila Rosamilia KANTOVITZ

**Affiliations:** (a) Faculdade São Leopoldo Mandic, School of Dentistry, Campinas, SP, Brazil.; (b) University of Saskatchewan, Dental School, Department of Operative Dentistry, Saskatoon, SK, Canada.; (c) Universidade Estadual de Campinas – Unicamp, Piracicaba Dental School, Department of Restorative Dentistry, Piracicaba, SP, Brazil.; (d) Universidade Estadual Paulista – Unesp, Dental School, Department of Health Sciences and Pediatric Dentistry, São Paulo, SP, Brazil.; (e) Universidade Estadual Paulista – Unesp, School of Science, Department of Physics, Bauru, SP, Brazil.

**Keywords:** Nanotechnology, Glass Ionomer Cement, Titanium

## Abstract

The present in vitro study evaluated the effect of titanium dioxide nanotubes (nTiO_2_) incorporated into glass ionomer cement (GIC) on the growth and viability of *Lactobacillus acidophilus* (L. acidophilus). GIC (Ketac Molar EasyMix^®^ = KM) was added to concentrations of 0%, 3%, 5%, 7% by weight of nTiO_2_ (20 nm in size). L. acidophilus strains (1x10^8^ CFU/mL) were cultivated on GIC discs with or without the addition of nTiO_2_ for 1, 3 and 7 days, and the following parameters were evaluated: inhibition zone (mm) (n = 6); cell viability (Live/Dead) (n = 6); cell morphology (SEM) (15 KV, 2000X, n = 3). The data were submitted to ANOVA and the Tukey and Dunnett tests (α = 0.05). Regarding the agar diffusion test, there was no difference between GIC and the groups containing nTiO_2_ (p > 0.05). As for bacterial viability, the percentage of viable bacteria was lower for GIC+7% nTiO_2_ (p ≤ 0.05). There was no difference in the percentage of non-viable bacteria (p > 0.05). In addition, the morphology of L. acidophilus did not change in the presence of nTiO_2_. It can be concluded that the incorporation of titanium dioxide nanotubes into GIC, particularly at 5%, reduced *L. acidophilus* viability, and might hence interfere negatively with the initial colonization process of the bacterial biofilm.

## Introduction

Dental biofilm is a complex microbial ecosystem consisting of a three-dimensional matrix, developed from multiple bacterial species.^1^ The accumulation of this bacterial mass on restorative materials facilitates their deterioration, microleakage formation between tooth and restoration, and the development of carious lesions in areas adjacent to the restorations.^2^ Several species of bacteria have been isolated from the dental biofilm associated with carious lesions, among which *Lactobacillus* stands out as one of the pathogens with the greatest involvement in the progression of active lesions of dental caries in children and adults.^3,4^

There is a growing interest in materials capable of modulating the organization and maturation of dental biofilm, particularly conventional glass ionomer cement (GIC), aiming to prevent or delay carious lesion progression, deterioration of the restorative material surface, and consequent reduction in the rate of restoration replacement.^2,5-9^ Some of the properties of GIC include chemical adhesion to the tooth structure, coefficient of linear thermal expansion close to the dentin, and antimicrobial properties derived from the release of fluoride between 2 and 10 ppm in the first 48 h or less, for a prolonged period.^8,10^ However, this material has certain limitations regarding high masticatory effort, owing to its low cohesive strength.^11,12^

Striving to improve the mechanical properties of GIC, researchers have established that the incorporation of titanium dioxide (TiO_2_) nanoparticles results in a significant increase in compressive strength, surface microhardness, solubility, color opacity and radiopacity, as well as fluorine release levels.^13-18^ Furthermore, since TiO_2_ decomposes organic compounds through the formation and constant release of hydroxyl radicals and superoxide ions, it is highly efficient in inhibiting bacterial growth.^19,20^

In this context, few studies in the literature have evaluated the impact of incorporating nanotechnology into the GIC surface on bacterial biofilm formation. These studies investigated the effect of TiO_2_ nanoparticles, alone or in association with chitosan, hydroxyapatite or sisal cellulose incorporated into restorative materials on Streptococcus mutans and Candida albicans.^13,14,21-23^Additionally, the structural differences among the various nanomaterials (e.g., nanoparticles, nanotubes, nanowires, nanorods, and nanofilms) have been proposed as the key mechanical-biological property of GIC.^20^ The shape of nanotubes yields a high surface area to volume ratio, believed to facilitate loading force dissipation, by providing optimal dispersion and surface energy.^18^ Therefore, the pioneering objective of the present in vitro study was to evaluate the impact of incorporating different concentrations of TiO_2_ nanotubes (nTiO_2_) into GIC on the biology of *Lactobacillus acidophilus* cultures at the cellular level. Thus, the null hypothesis of the present investigation was that nTiO_2_ added to conventional type IV ionomer (GIC) does not affect the biology of *L. acidophilus* during the initial periods of biofilm formation, including cell viability and morphology.

## Methods

### Experimental design

The present in vitro study evaluated the response of *L. acidophilus* (ATCC 4356) cultured on high-viscosity conventional GIC (Ketac Molar EasyMix-3M/ESPE, Maplewood, USA, lots #635287 and #610966) incorporated with different concentrations of nTiO_2_ (0, 3, 5 and 7% by weight) at different time points of maturation of the biofilm formed on the material (1, 3 and 7 days). GIC specimens were randomly distributed into four experimental groups: GIC = Control; GIC+3% nTiO_2_; GIC+5% nTiO_2_; and GIC+7% nTiO_2_ (n = 6/group) according to the study by Kantovitz et al.^18^ The following parameters were evaluated: a) Agar diffusion test (mm) (n = 6); b) Cell viability (CFU/mL) (n = 6); and c) Cell morphology (scanning electron microscope) (n = 3). The experiments were performed in triplicate and repeated at least twice, according to ISO 10993-5 (2009) recommendations.^23^

### Materials

nTiO_2_ (particle size ~20 nm, and diameter ~10 nm) was synthesized by the alkaline method, as described earlier.^23^ A conventional high-viscosity GIC, Ketac Molar EasyMix^TM^ (shade A3; powder: Al-Ca-La fluorosilicate glass, 5% copolymer acid [acrylic and maleic acid] [15 g]; liquid: acrylic acid-maleic acid copolymer [25–40% by weight], tartaric acid [5–10% by weight], and water [10 g]), was used based on a previous review.^24^

### Sample preparation

The nTiO_2_ was weighed on a precision scale, accurate to 0.0001 g (Adventurer Ohaus, Parsippany, USA), and added to the powder component of KM, after which it was homogenized vigorously in a QL-901 vortex mixer (BioMixer, Taft, USA) for 2 minutes.^18^ The recommended powder/liquid (P/L) ratio of 2/2 for GIC was used for all the specimens prepared, and manipulation followed the manufacturer’s specifications. GIC and GIC+nTiO_2_ discs were prepared using Teflon split disc molds filled in one increment using a Centrix syringe, and then pressed for 6 minutes between the polyester matrix (Proben, Catanduva, Brazil, #PR5021) and glass plates with a 200 g weight. After the initial setting time was completed, the specimens were covered with a thin layer of petroleum jelly (Rioquímica, São José do Rio Preto, Brazil, #1702146) and stored for 24 h (final setting) at 37°C and 100% humidity. The discs were exposed to ultraviolet light for 15 minutes on each surface before initiating the experiments.^16,18^

### *Lactobacillus acidophilus* cultures

*L. acidophilus* strains (ATCC 4356) were used in the present study. For each experiment, 300 µL of the frozen stock were freshly cultured in 15 mL Falcon-type tubes with 5 mL of Man-Rogosa-Sharpe (MRS) (DIFCO Laboratories, Detroit, USA). The absorbance of 0.135 at 660 nm was achieved to obtain a concentration of 1x10^8^ cells/mL (Genesys 2 spectrophotometer, Spectronic Unicam, Waltham, USA), using the MacFarland scale.^23^ The cultures were incubated for 24 h at 37°C in 10% CO_2_ for subsequent experiments.

### Agar diffusion test

A base layer was prepared for each Petri dish (15 x 90 mm), containing 15 mL of sterile MRS medium mixed with the inoculum. After MRS agar solidification, six wells measuring 5 mm in diameter were prepared in each plate and filled with one of the experimental materials (GIC with or without nTiO_2_). All the materials were handled under aseptic conditions. A thin layer of the agar was added to the wells, allowing total involvement of the material in the culture medium. Chlorhexidine digluconate 0.12% solution and sterilized deionized water (10 μL) were applied on sterile filter paper disks, as a positive and negative control, respectively. The dishes were kept for 2 h at room temperature to allow diffusion of the materials, and then incubated at 37°C and 10% CO_2._^23^ After 1, 3 and 7 days of incubation, the inhibition zones around the materials were measured (mm) by a calibrated evaluator (Spearman correlation = 83%) using a digital caliper (Mitutoyo, MTI Corporation, Tokyo, Japan).

### Cell viability test (Live/Dead)

*L. acidophilus* were cultured on GIC disks with or without nTiO_2_ (2 mm in height x 4 mm in diameter) in 24-well plates for 1, 3 and 7 days (37°C and 10% CO_2_). The culture medium was replaced every 48 h to maintain viable microorganism growth. The culture medium was aspirated, and the discs were washed with saline solution. The plates were kept in a dark room, and 25 µL of a Live/Dead BacLight bacterial viability stain solution was applied (Molecular Probes, Eugene, USA). The excitation/emission wavelengths of SYTO9 and propidium iodide were 480/500 nm and 490/635 nm, respectively. Six images were captured in a fluorescence microscope (Zeiss, Jena, GER) with 400X magnification from randomly selected sites for each surface analyzed. The green and red zones, representing live and dead bacterial cells, respectively, were assessed separately to determine the viability of the adhered bacterial species for each type of surface treatment. The bacterial cell count for each dye in relation to the total area was performed using ImageJ software (National Institute of Health, NIH, Bethesda, USA) and presented in arbitrary units (a.u.) and percentages for each surface. All the images had a standard area of 97 µm^2^, totaling 582 µm^2^ for the six images analyzed. Positive and negative controls comprised the bacteria grown on glass coverslips, and cultures treated with chlorhexidine digluconate 0.12% solution, respectively.

### Cell morphology by Scanning Electron Microscopy (SEM)

*L. acidophilus* strains were cultivated as described in the previous item. The cells were fixed in Karnovsky’s solution (2.5% glutaraldehyde and 2.0% paraformaldehyde in 0.1 M phosphate buffer, pH 7.4) for 2 h at room temperature. Subsequently, the specimens were dehydrated in a graded series of ethanol solutions, and then stored in a lab oven at 37°C for overnight dehydration.^16,23^Next, the specimens were dried to the critical point (Denton Vacuum, mod. DCP-1, Moorestown, USA), sputter-coated with a 10-nm layer of gold (BAL-TEC, model SCD 050, Fürstentum, Liechtenstein), and kept in a desiccator until analysis. Cell structure and morphology of *L. acidophilus* strains were evaluated by a JEOL SEM (JSM5600LV, Akishima, Tokyo, Japan) at 2000X magnification, 15 kV and 10 mm working distance.

### Statistical analysis

Data distribution and homoscedasticity were analyzed by Shapiro-Wilk and Levene tests, respectively (p≥0.05). Inhibition halo data were subjected to repeated measures ANOVA, followed by Tukey’s test for multiple comparisons, whereas Dunn’s test was used to assess statistical differences between the control and experimental groups individually. In addition, cell viability data (viable, non-viable and total bacteria) were converted with Box-Cox, and the two-way ANOVA and Tukey’s tests were performed. Cell morphology was evaluated descriptively. Statistical analyses were performed using SPSS Statistics (version 21, IBM Statistics, New York, USA) and SAS System (Cary, USA) software (α = 0.05).

## Results

Table presents data from the agar diffusion test in *L. acidophilus* cultures over time. There was no significant difference between the GIC and the groups containing nTiO_2_ within each time point studied (p ≥ 0.05). There was an increase within each experimental group in the inhibition zone on the 3^rd^ and 7^th^ days, which were statistically different from the 1^st^ day (p ≥ 0.05).

**Table:** Mean (standard deviation) diameter of the inhibition zone (mm) according to the experimental groups over time (1, 3 and 7 days) for *L. acidophilus* (n = 6).

Bacterial strain	Experimental group	Day 1	Day 3	Day 7
L. acidophilus	GIC	7.32 (0.48) Ab	7.82 (0.28) Aa	7.79 (0.31) Aa
	GIC + 3% nTiO_2_	7.19 (0.19) Ab	7.67 (0.20) Aa	7.83 (0.46) Aa
	GIC + 5% nTiO_2_	7.69 (0.40) Ab	7.78 (0.24) Aa	7.55 (0.24) Aa
	GIC + 7% nTiO_2_	7.83 (0.45) Ab	7.93 (0.22) Aa	7.86 (0.26) Aa
	CHX	13.57 (0.56) ***	13.45 (0.39)***	13.69 (0.52) ***

Different letters indicate a statistically significant difference using the repeated measures ANOVA test with additional treatment (Dunnett’s test). Uppercase letters indicate intergroup statistical differences (within the same experimental period) and lowercase letters indicate intragroup statistical differences (p < 0.05). GIC = Ketac Molar EasyMix; nTiO_2_ = titanium dioxide nanotubes; CHX = chlorhexidine 0.12%.

**Figure 1:**
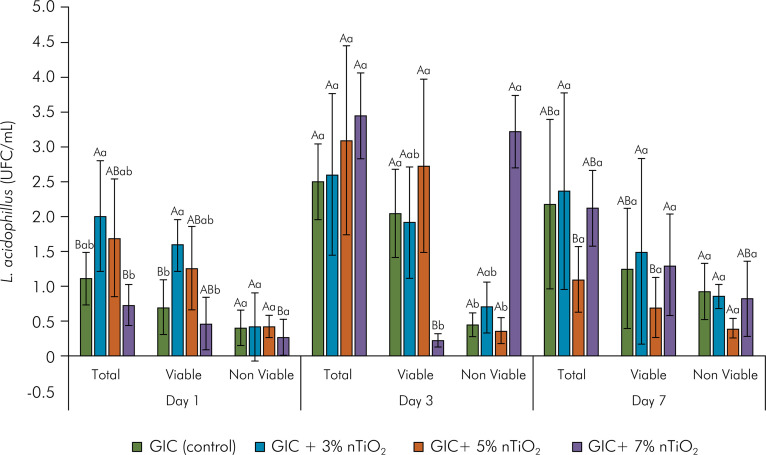
Mean (standard deviation) of the number of bacteria (total, viable and non-viable *L. acidophilus* - CFU/mL) according to the nTiO2 concentration (0, 3, 5 and 7% in weight), and to the biofilm maturation time (1, 3 and 7 days) (n = 6/group).

**Figure 2:**
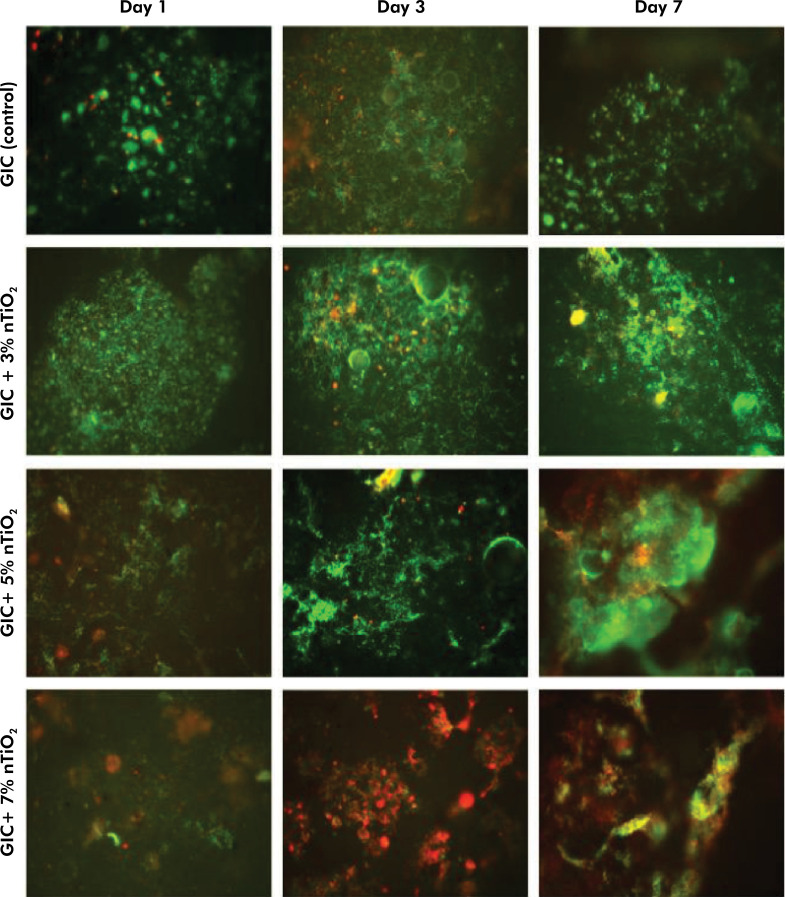
Representative fluorescence microscopy images of the Live/Dead assay according to nTiO2 concentration (0, 3, 5 and 7% by weight) and biofilm maturation time (1, 3 and 7 days) (n = 6/group).

Cell viability data, average number of total and viable bacteria (CFU/mL) according to nTiO_2_ concentration, and time (1, 3 and 7 days) of biofilm maturation are shown in Figure 1 and Figure 2. Overall, the total number of *L. acidophilus* bacteria increased according to the biofilm maturation time, except for the biofilm formed on GIC+5% nTiO_2_, which showed a decrease in the colony-forming units on the 7^th^ day (p = 0.0254). In the intragroup analysis, GIC containing 7% nTiO_2_ within the same experimental time (Day 1) significantly reduced the number of total bacterial colonies per milliliter (p = 0.0048), whereas the concentration of nTiO_2_ incorporated into GIC on days 3 and 7 of *L. acidophilus* biofilm maturation did not modify the antimicrobial action of GIC (p = 0.2025 and p = 0.0820, respectively) (Figure 1). Furthermore, GIC with no added nanotechnology showed the lowest amount of bacterial biofilm (p = 0.0027). On Day 3, GIC incorporated with 7% nTiO_2_ stands out as having a lower number of viable *L. acidophilus* (p=0.0024), with no statistical difference among the groups on Day 7 of biofilm maturation (p = 0.7708) (Figure 1). Additionally, Figure 2 presents a qualitative analysis of the fluorescence microscopy images, representing the live (green) and dead (red) cells in contact with the materials at different time points. Fluorescence images are divided into rows (groups) and columns (time points).

Different uppercase letters indicate a statistically significant difference according to biofilm maturation time (intragroup) (p ≤ 0.05). Different lowercase letters indicate a statistically significant difference according to nTiO_2_ concentration (intergroup) within the same experimental time point (p = 0.002); GIC: Ketac Molar EasyMix; nTiO_2_: titanium dioxide nanotubes.

The qualitative analysis shows the viable (in green) and non-viable (in red) bacteria in contact with the materials at different time points. Fluorescence images are divided into rows (groups) and columns (time points).

**Figure 3:**
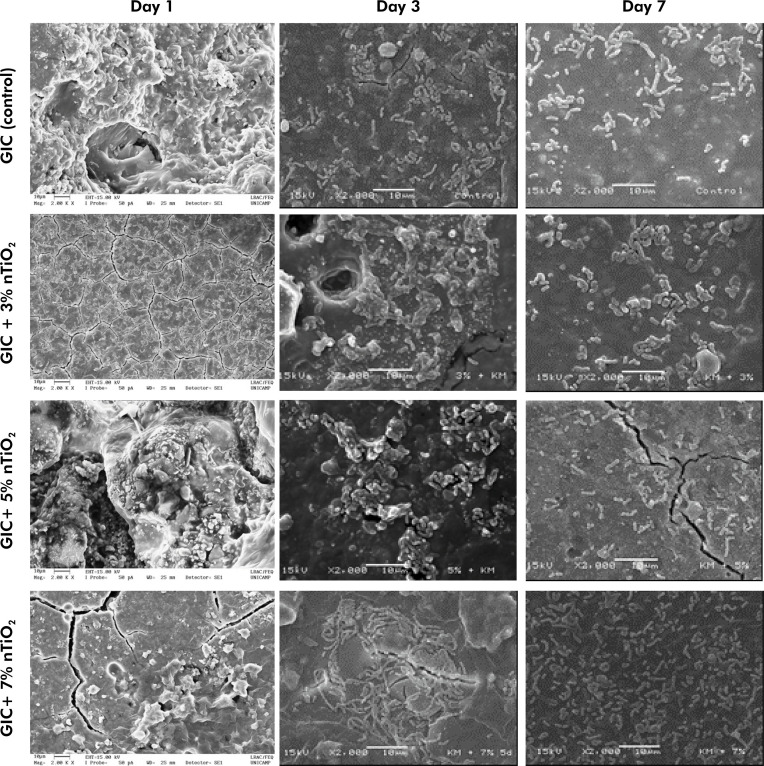
Representative scanning electron microscopy images (2000X) of *L. acidophilus* strain morphology, according to nTiO2 concentration (0, 3, 5 and 7% by weight) and biofilm maturation time (1, 3 and 7 days) (n = 3/group).

Figure 3 shows representative SEM images of the morphology of *L. acidophilus* cultivated on GIC discs with or without the incorporation of nTiO_2_, at 2000X magnification, according to the experimental time points (days 1, 3 and 7). Data shows that nTiO_2_ did not affect the cell morphology, thereby indicating a typical aspect of rods with rounded ends, between 1.5 and 3.0 m in length, presented as single cells, or cells in pairs or in chains.

SEM images are divided into rows (groups) and columns (time points). Red arrows indicate the typical aspect of rods with rounded end single cells, while white arrows indicate cells in pairs or in chains. GIC: Ketac Molar EasyMix; nTiO2: titanium dioxide nanotubes.

## Discussion

In the present study, the findings of the agar inhibition halo assay showed that there was no significant difference between GIC and the groups with nTiO_2_, at any of the studied time periods (p ≥ 0.05), for the *L. acidophilus* groups, nor did nTiO_2_ affect cell morphology. As a result, the cells had a typical rod-like appearance with rounded ends. Therefore, the first hypothesis of the present study failed to be rejected. It is noteworthy to mention that the inhibition halo measuring 7.32 ± 0.48 mm in the control group, without the addition of nanotechnology on Day 1, is consistent with that described in the literature.^25,26,7^ However, the findings of the present study differed from those of previous in vitro studies that have suggested a potential antimicrobial effect on S. mutans and/or Candida albicans for conventional GICs supplemented with TiO_2_ nanostructures, alone or in association with chitosan, hydroxyapatite, silver and/or sisal cellulose incorporated into restorative materials.^13,14,23,27,28^ The nanoparticles tend to agglomerate within the restorative material, thereby altering their biological properties.^18,20^ Bear in mind, however, that this halo inhibition method is often used to assess the antimicrobial activity of dental materials, because it is practical and ideal for fast-growing bacteria.^13,29^ It must be considered that any numerical comparison of different studies must be carried out with caution, since the methodologies may differ in terms of the density of the cultured inoculum, size of the Petri dish used, and inoculation method, all of which can influence the zone of inhibition.^23^ A large amount of inoculum shortens the critical time, and can result in a falsely smaller zone of inhibition, whereas a small amount of inoculum causes the reverse effect, and brings about a falsely larger zone of inhibition. The present study used 300 L of inoculum, while the study by Naik et al.^7^used 500 L, perhaps explaining the difference in the inhibition zones.

The second null hypothesis of the present study was rejected, since the cell viability assay (live/dead) demonstrated that as the nTiO_2_ concentration increases, the number of viable *L. acidophilus* bacteria drops on Day 1. On Day 7 of the evaluation, the concentration of 5% nTiO_2_ showed the lowest number of viable bacteria (p = 0.0027) (Figure 1). These findings corroborate those of previous studies, which proved the bactericidal power of TiO_2_, used pure or added to dental materials, in inactivating several microorganisms involved with diseases of the oral cavity.^13,20-22,30,31^ Although the mechanisms of the potential antibacterial effect of TiO_2_ nanostructures have not yet been fully elucidated, it has been suggested that electrostatic interactions between metal ions of nanostructured titanium and the bacteria^32^ that bind to the cell membrane,^30^ together with the effects of these interactions on phospholipids,^33^ may explain some of the events that control bacterial viability in the presence of nTiO_2_.

There are indications that bacteria are less prone to develop resistance to metal nanostructures than to antibiotics. This is because metals may disturb different cellular processes, thus requiring diverse adaptive mechanisms for bacteria to acquire resistance.^34^ Although an increase in the concentration of nanostructures added to GIC can lead to an increase in antibacterial performance, the concentration of 7% TiO_2_ added to the conventional GIC, as used in the present study, had a result similar to that of the control group (with no added nanotechnology) on Day 7 of biofilm maturation. A possible explanation for the antibacterial action of nTiO_2_ incorporated into conventional GICs might be its effect on fluoride release, as demonstrated by previous studies at 5% nTiO_2_.^16,35^ In the current study, GIC containing 5% nTiO_2_ significantly affected *L. acidophilus* viability, and may serve as a reference to guide future research assessing the antibacterial effect of n-TiO_2_ added to restorative dental material. In addition to its antimicrobial activity, the concentration of 5% nTiO₂ in GIC also enhances other critical properties of the material, such as compressive strength, surface microhardness, and solubility of the GIC, without altering its surface roughness.^16-18,35^ Using this concentration would prevent unnecessary fractures of the restoration and infiltration of bacteria, or could even serve as a substrate for bacteria, thus contributing to the longevity of the restoration.

It has also been suggested that the specific physicochemical properties of nanostructures may play a critical role in their functionality and use.^20^ The antimicrobial action of TiO_2_ is related to its use in different sizes in the nanometer scale (20–80 µm), and different formats (spherical, wire, particle, tube), and also to the crystallinity of the structure used.^20,36,37^ Regarding TiO_2_ crystallinity—determined by the type of crystal synthesized—there are three different forms: anatase, rutile, and brookite. The first has been identified as affording the greatest antibacterial activity. This warrants its use in the present study. A study in the literature reports that during nanotube formation, structures in the anatase phase produce OH^-^ ions as a photocatalytic reaction; these structures perforate the bacterial membrane and lead to its death.^30^

In addition to the potential direct effect of nTiO_2_ on bacterial survival, as discussed hereinabove, previous studies have shown that GIC incorporated at 5% nTiO_2_ intensifies the material’s fluoride release, and reduces aluminum release rates,^16,35^ thus influencing the antibacterial ability of GIC. Considering the morphology of *L. acidophilus* cultures, the current findings demonstrated that the incorporation of nTiO_2_ into GIC did not alter the morphology of these bacteria. Furthermore, there are no existing studies for comparison with our findings.

In vitro biofilm models are widely used to evaluate new formulations.^38^ Even though valid results were obtained in the present study, the data generated may be limited in terms of revealing the true behavior of the product in in vivo studies. However, despite the relative simplicity of the study results, the data can establish and evaluate the efficacy of these models in the experimentation phase of new restorative formulations before performing clinical studies.^38^ In this regard, although in vitro models of biofilms are far from representing the complex biofilms that exist in the oral cavity, they could eventually establish the release profile for this nanotechnology. The importance of the present study lies in its aim to shed light on innovative technology, and to join continuous, integrated efforts of basic research to aid in launching a new restorative material.

Based on the profile of GIC behavior when incorporated into nTiO_2_ in the presence of a monospecies biofilm, future studies should be conducted to understand the mechanism of action of this nanotechnology, and determine its action in the bacterial mutation of genes. In addition, live in-depth investigations of widely used dental material should be carried out to supplement this information.

## Conclusion

It can be concluded that the incorporation of titanium dioxide nanotubes into GIC, particularly at 5%, reduced *L. acidophilus* viability, and might hence interfere negatively with the initial colonization process of the bacterial biofilm.

## References

[1] Karygianni L, Ren Z, Koo H, Thurnheer T (2020). Biofilm matrixome: extracellular components in structured microbial communities. Trends Microbiol.

[2] Busscher HJ, Rinastiti M, Siswomihardjo W, Mei HC (2010). Biofilm formation on dental restorative and implant materials. J Dent Res.

[3] Callaway A, Kostrzewa M, Willershausen B, Schmidt F, Thiede B, Küpper H (2013). Identification of Lactobacilli from deep carious lesions by means of species-specific PCR and MALDI-TOF mass spectrometry. Clin Lab.

[4] Caufield PW, Schön CN, Saraithong P, Li Y, Argimón S (2015). Oral lactobacilli and dental caries: a model for niche adaptation in humans. J Dent Res.

[5] Khademolhosseini MR, Barounian MH, Eskandari A, Aminzare M, Zahedi AM, Ghahremani D (2012). Development of new Al _2_ O _3_/TiO _2_ reinforced glass-ionomer cements (GICs) nano-composites. J Basic Appl Sci Res.

[6] Farrugia C, Camilleri J (2015). Antimicrobial properties of conventional restorative filling materials and advances in antimicrobial properties of composite resins and glass ionomer cements: a literature review. Dent Mater.

[7] Naik RG, Dodamani AS, Khairnar MR, Jadhav HC, Deshmukh MA (2016). Comparative assessment of antibacterial activity of different glass ionomer cements on cariogenic bacteria. Restor Dent Endod.

[8] Fúcio SB, Paula AB, Sardi JC, Duque C, Correr-Sobrinho L, Puppin-Rontani RM (2016). Streptococcus Mutans Biofilm influences on the antimicrobial properties of glass ionomer cements. Braz Dent J.

[9] Hafshejani TM, Zamanian A, Venugopal JR, Rezvani Z, Sefat F, Saeb MR (2017). Antibacterial glass-ionomer cement restorative materials: A critical review on the current status of extended release formulations. J Control Release.

[10] Anusavice KJ, Shen C, Rawls HR (2013). Phillips' science of dental materials.

[11] Menezes-Silva R, Cabral RN, Pascotto RC, Borges AF, Martins CC, Navarro MF (2019). Mechanical and optical properties of conventional restorative glass-ionomer cements: a systematic review. J Appl Oral Sci.

[12] Nicholson JW, Coleman NJ, Sidhu SK (2021). Kinetics of ion release from a conventional glass-ionomer cement. J Mater Sci Mater Med.

[13] Elsaka SE, Hamouda IM, Swain MV (2011). Titanium dioxide nanoparticles addition to a conventional glass-ionomer restorative: influence on physical and antibacterial properties. J Dent.

[14] Garcia-Contreras R, Scougall-Vilchis RJ, Contreras-Bulnes R, Sakagami H, Morales-Luckie RA, Nakajima H (2015). Mechanical, antibacterial and bond strength properties of nano-titanium-enriched glass ionomer cement. J Appl Oral Sci.

[15] Najeeb S, Khurshid Z, Zafar MS, Khan AS, Zohaib S, Martí JM (2016). Modifications in glass ionomer cements: nano-sized fillers and bioactive nanoceramics. Int J Mol Sci.

[16] Cibim DD, Saito MT, Giovani PA, Borges AF, Pecorari VG, Gomes OP (2017). Novel nanotechnology of TiO 2 improves physical-chemical and biological properties of glass ionomer cement. Int J Biomater.

[17] Kantovitz KR, Fernandes FP, Feitosa IV, Lazzarini MO, Denucci GC, Gomes OP (2020). TiO 2 nanotubes improve physico-mechanical properties of glass ionomer cement. Dent Mater.

[18] Kantovitz KR, Carlos NR, Silva IA, Braido C, Costa BC, Kitagawa IL (2023). TiO 2 nanotube-based nanotechnology applied to high-viscosity conventional glass-ionomer cement: ultrastructural analyses and physicochemical characterization. Odontology.

[19] Shah MS, Nag M, Kalagara T, Singh S, Manorama SV (2008). Silver on PEG-PU-TiO^2^ polymer nanocomposite films; an excellent system for antibacterial applications. Chem Mater.

[20] Vimbela GV, Ngo SM, Fraze C, Yang L, Stout DA (2017). Antibacterial properties and toxicity from metallic nanomaterials. Int J Nanomedicine.

[21] Ibrahim MA, Meera Priyadarshini B, Neo J, Fawzy AS (2017). Characterization of Chitosan/TiO 2 nano-powder modified glass-ionomer cement for restorative dental applications. J Esthet Restor Dent.

[22] Sun J, Xu Y, Zhu B, Gao G, Ren J, Wang H (2019). Synergistic effects of titanium dioxide and cellulose on the properties of glassionomer cement. Dent Mater J.

[23] Araújo IJ, Ricardo MG, Gomes OP, Giovani PA, Puppin-Rontani J, Pecorari VA (2021). Titanium dioxide nanotubes added to glass ionomer cements affect S. mutans viability and mechanisms of virulence. Braz Oral Res.

[24] Amorim RG, Frencken JE, Raggio DP, Chen X, Hu X, Leal SC (2018). Survival percentages of atraumatic restorative treatment (ART) restorations and sealants in posterior teeth: an updated systematic review and meta-analysis. Clin Oral Investig.

[25] Duque C, Negrini TC, Hebling J, Spolidorio DM (2005). Inhibitory activity of glass-ionomer cements on cariogenic bacteria. Oper Dent.

[26] Silva IA, de Souza Araújo IJ, Stipp RN, Puppin Rontani RM (2020). Glass-ionomer cement modifies the gene expression of Streptococcus mutans providing a lower virulent biofilm. Am J Dent.

[27] Sodagar A, Akhavan A, Hashemi E, Arab S, Pourhajibagher M, Sodagar K (2016). Evaluation of the antibacterial activity of a conventional orthodontic composite containing silver/hydroxyapatite nanoparticles. Prog Orthod.

[28] Mahendra TV, Muddada V, Gorantla S, Karri T, Mulakala V, Prasad R (2022). Evaluation of antibacterial properties and shear bond strength of orthodontic composites containing silver nanoparticles, titanium dioxide nanoparticles and fluoride: an in vitro study. Dental Press J Orthod.

[29] de Paula AB, Fucio SB, Ambrosano GM, Alonso RC, Sardi JC, Puppin-Rontani RM (2011). Biodegradation and abrasive wear of nano restorative materials. Oper Dent.

[30] Zhou Y, Kong Y, Kundu S, Cirillo JD, Liang H (2012). Antibacterial activities of gold and silver nanoparticles against Escherichia coli and bacillus Calmette-Guérin. J Nanobiotechnology.

[31] Jesline A, Neetu PJ, Narayanan PM, Vani C, Sevanan M (2015). Antimicrobiaal activity of zinc and titanium dioxide nanoparticles against biofilm-prodicing methicillin-resistant Staphylococcus aureus. Appl Nanosci.

[32] Tavassoli Hojati S, Alaghemand H, Hamze F, Ahmadian Babaki F, Rajab-Nia R, Rezvani MB (2013). Antibacterial, physical and mechanical properties of flowable resin composites containing zinc oxide nanoparticles. Dent Mater.

[33] Wong MS, Chu WC, Sun DS, Huang HS, Chen JH, Tsai PJ (2006). Visible-light-induced bactericidal activity of a nitrogen-doped titanium photocatalyst against human pathogens. Appl Environ Microbiol.

[34] Pal S, Tak YK, Song JM (2007). Does the antibacterial activity of silver nanoparticles depend on the shape of the nanoparticle? A study of the Gram-negative bacterium Escherichia coli. Appl Environ Microbiol.

[35] Morais AM, Pereira YM, Souza-Araújo IJ, Silva DF, Pecorari VG, Gomes OP (2022). TiO 2 nanotube-containing glass ionomer cements display reduced aluminum release rates. Braz Oral Res.

[36] Ercan B, Taylor E, Alpaslan E, Webster TJ (2011). Diameter of titanium nanotubes influences anti-bacterial efficacy. Nanotechnology.

[37] Ashkarran AA, Hamidinezhad H, Haddadi H, Mahmoudi M (2014). Double- doped TiO_2_ nanoparticles as an efficient visible-light-active photo- catalyst and antibacterial agent under solar simulated light. Appl Surf Sci.

[38] Omar A, Nadworny P (2017). Review: Antimicrobial efficacy validation using in vitro and in vivo testing methods.

